# A Method Integrating the Matching Field Algorithm for the Three-Dimensional Positioning and Search of Underwater Wrecked Targets

**DOI:** 10.3390/s25154762

**Published:** 2025-08-01

**Authors:** Huapeng Cao, Tingting Yang, Ka-Fai Cedric Yiu

**Affiliations:** 1 School of Navigation, Dalian Maritime University, Dalian 116026, China; vistachris@163.com (H.C.); yangtingting820523@163.com (T.Y.); 2 Peng Cheng Laboratory, Shenzhen 518066, China; 3 Department of Applied Mathematics, The Hong Kong Polytechnic University, Hong Kong 999077, China

**Keywords:** maritime search, direction of arrival estimation, matched field processing, three-dimensional positioning, beamforming

## Abstract

In this paper, a joint Matching Field Processing (MFP) Algorithm based on horizontal uniform circular array (UCA) is proposed for three-dimensional position of underwater wrecked targets. Firstly, a Marine search and rescue position model based on Minimum Variance Distortionless Response (MVDR) and matching field quadratic joint Algorithm was proposed. Secondly, an MVDR beamforming method based on pre-Kalman filtering is designed to refine the real-time DOA estimation of the desired signal and the interference source, and the sound source azimuth is determined for prepositioning. The antenna array weights are dynamically adjusted according to the filtered DOA information. Finally, the Adaptive Matching Field Algorithm (AMFP) used the DOA information to calculate the range and depth of the lost target, and obtained the range and depth estimates. Thus, the 3D position of the lost underwater target is jointly estimated. This method alleviates the angle ambiguity problem and does not require a computationally intensive 2D spectral search. The simulation results show that the proposed method can better realise underwater three-dimensional positioning under certain signal-to-noise ratio conditions. When there is no error in the sensor coordinates, the positioning error is smaller than that of the baseline method as the SNR increases. When the SNR is 0 dB, with the increase in the sensor coordinate error, the target location error increases but is smaller than the error amplitude of the benchmark Algorithm. The experimental results verify the robustness of the proposed framework in the hierarchical ocean environment, which provides a practical basis for the deployment of rapid response underwater positioning systems in maritime search and rescue scenarios.

## 1. Introduction

Ocean exploration and maritime trade activities are increasingly frequent, and accidents involving ships, submarines, underwater robots, and other marine targets may result in sinking. Accidents at sea not only lead to property damage but also cause many casualties. Therefore, it is essential to search for sunken targets in the ocean. However, the difficulty of maritime search and rescue operations is greater than on land, mainly due to the uncertainty of the location of the accident, the large search area, limitations in search technology, harsh underwater survival conditions, weather changes, and seawater movements. This further highlights the importance of search technology. After an accident, the last known position coordinates of the target become the key to the search. The three-dimensional positioning of underwater targets is a crucial technology, including estimating three parameters, angle, distance, and depth, to determine the precise location and direction of the underwater target. Beamforming is a signal processing technique that enhances signals from a specific direction and suppresses noise from other directions by applying specific weights on a sensor array to form a beam pointed in that direction. Common methods include conventional beamforming (CBF) [1] and Minimum Variance Distortionless Response (MVDR) [2]. Direction of Arrival (DOA) estimation is based on beamforming results to accurately estimate the direction, distance, and depth of the signal source. Distance and depth parameters can be estimated using the Matched Field Processing (MFP), with common methods including the Conventional Matched Field Processing (CMFP) [3] and the Adaptive Matched Field Processing (AMFP) [4]. These technologies are crucial for improving the accuracy and efficiency of underwater search and rescue positioning, enabling search and rescue teams to quickly locate sunken ships, aircraft wreckage, or other important targets in the vast ocean environment.

In underwater DOA estimation, the Uniform Linear Array (ULA) is widely used for one-dimensional DOA estimation due to its simple structure and relatively small computational load. However, the ULA has the disadvantage of potential ambiguity in azimuth estimation and resolution that is limited by the element spacing and wavelength, leading to an inability to distinguish adjacent signal sources accurately in some cases. In contrast, the uniform circular array (UCA) can provide 360° full-coverage and unambiguous azimuth estimation, offering approximately the same resolution in all directions. The UCA can simultaneously estimate two-dimensional angles for both azimuth and elevation angles, offering greater superiority and practical value. However, in the field of array signal processing, UCA is difficult to apply conventional methods directly due to its complex steering vector, which is different from the Vandermonde structure of ULA. Various innovative methods have been proposed in the paper to address this challenge. The paper [5] first introduced the concept of paper pattern space transformation, laying the foundation for subsequent research by constructing a specific transformation matrix to convert the UCA into a virtual ULA with a Vandermonde structure. Building on this, the paper [6] developed the UCA-RB-MUSIC and UCA-ESPRIT algorithms, which use real-valued beamforming transformation matrices to estimate the two-dimensional angle information of signals using the MUSIC and ESPRIT algorithms. However, these methods have limitations in handling coherent signals. To overcome this limitation, the paper [7] adopted strategies of array translation and subarray partitioning to solve the coherence problem and performed signal angle estimation through a two-dimensional spectral peak search, which has a large computational load and is difficult to meet the needs of real-time processing. The paper [8] improved resolution and real-time performance through Toeplitzization and matrix reconstruction techniques, although it can only provide one-dimensional angle information. Further research, such as the paper [9], used a sparse reconstruction model-based method to estimate the two-dimensional direction of coherent signals, considering that the objective function requires sparsity in the spatial dimension but not in the temporal dimension and used second-order cone programming to solve the convex optimisation problem and obtain the two-dimensional spatial spectrum, employing singular value decomposition to reduce computational complexity. Although this method is highly accurate and insensitive to coherent signals, it involves complex mathematical optimisation problems, and the solution process is quite challenging.

In terms of range and depth measurement, the appearance of MFP technology has improved the accuracy of underwater positioning. Fizell et al. [10] first utilised the MFP method for long-distance localisation of sound sources in the Arctic Ocean and achieved good results, thereby gradually making MFP technology a hot topic in underwater acoustic signal processing research. Lei et al. [11] located the position of the sound source by matching the cross-correlation function output of signals received by two hydrophones. However, this method requires a large spacing between the two synchronised hydrophones. Combining the characteristics of sound intensity being very sensitive to changes in the depth and frequency of the sound source, Yang et al. proposed a method for estimating the depth and distance of the sound source in the deep sea using an unsynchronised vertical array [12]. Experimental results from the Western Pacific have shown that this method can locate fixed sound sources using an unsynchronised vertical array. To reduce the impact of reference sound speed on DOA estimation errors, a DOA estimation method independent of the reference sound speed is proposed [13]. In the field of matched field localisation, the design of arrays for matched field localisation and the positioning challenges in shallow water with non-uniform horizontal environments have been thoroughly examined [14]. Prof. Tang [15] proposed a distributed cooperative underwater node position Algorithm aided by factor graph weighted particles. The Algorithm uses factor graph sum and product Algorithm to decompose the global optimisation into the product of several local optimisation functions, and combines Gaussian parameters to construct weighted particles and realise belief transfer, which is suitable for underwater networks with limited energy and communication distance. Prof. Zhang [16] proposed a Gaussian parameterised information-aided distributed cooperative position Algorithm for underwater environments, considering the large signal propagation attenuation and the limited load of underwater nodes in the existing underwater position technology. The Algorithm used the Taylor model to linearly approximate the Euclidean distance. Then, combined with the factor mapping theory, the distributed cooperative position of underwater nodes was obtained. By combining the sum-product theory and parameterised Gaussian information passing, the communication computation is reduced.

Despite the extensive research on MFP methods, there are still many challenges in practical applications. For example, bathymetric maps provide the depth of the seafloor, but sunken targets, such as containers and debris, may rest on the seafloor due to partial burial or suspension. Their effective acoustic reflection depth is different from the seafloor depth, and direct depth estimation is required for accurate positioning. The most prominent issue is that the ocean channel is time-variant and space-variant, which leads to the simulated sound field and the measured sound field not being completely consistent, thereby affecting the performance of the matched field localisation Algorithm. Furthermore, errors in DOA estimation can affect the distance and depth assessment. In this paper, based on the summary of the above literature, we believe that the key point of maritime search and rescue localisation is underwater target localisation, which is essentially a three-dimensional localisation problem. We discuss the ability of a horizontal UCA to jointly estimate through beamforming technology and matching field localisation in three-dimensional localisation of underwater targets. Therefore, in this paper, we first analyse the basic principles of DOA estimation and matching field localisation Algorithm; a joint Algorithm based on MVDR combined with MFP based on pre-Kalman filtering (KF-MVDR-MF) is proposed by using UCA array, the MVDR beamforming method of Kalman filter, and the matching field Algorithm to obtain range and depth estimation to determine the 3D position of the sound source, which is used for the search and localisation of sunk targets in the ocean.

The rest of this paper is arranged as follows. Section 2 presents the mathematical model of ocean search and rescue system, analyses the basic principle of DOA estimation in Section 3, introduces some necessary background knowledge of matching field, and gives the signal waveform received by the AMFP Algorithm in the search and rescue scene in Section 4. We derive the 3D position joint beamforming Algorithm KF-MVDR-MF for underwater search and rescue position. Section 5 presents simulation results comparing our KF-MVDR-MF-based implementation with its original implementation. Finally, conclusions are drawn in Section 6.

## 2. System Model

**Figure 1:**
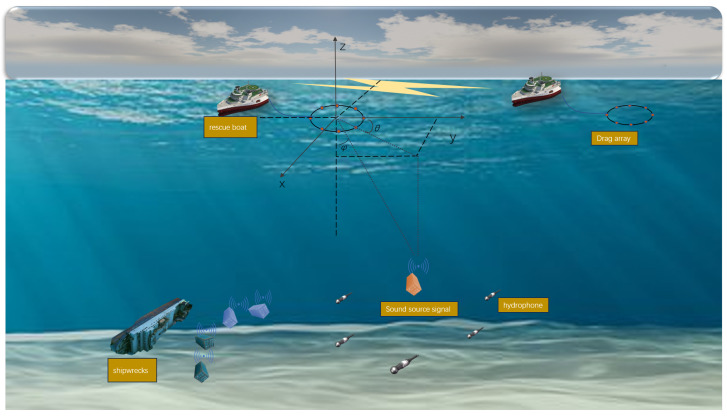
Search and rescue scene diagram.

We consider a search and rescue scenario where a large container ship has containers falling into the sea due to bad weather, resulting in *M* containers in the water. The accident site is located in the deep sea, requiring rescue and salvage departments to go to the incident area for positioning and salvage. The scenario described in this paper is shown in Figure 1. Azimuth angle ϕ denotes horizontal bearing; elevation angle θ defines vertical inclination.

It is assumed that each container is equipped with an underwater positioning beacon emitting a narrowband signal, with the beacon operating at 37.5 kHz. The search and rescue ship tows a uniform circular array with *N* elements to the incident area for search and rescue operations. The input of each element in the array is the sum of *M* signals and additive noise. After the boxes fall into the water, a delay water level switch is activated, and the signals received by the *N* elements can be arranged into a column vector to form a narrowband far-field signal direction estimation (DOA) mathematical model. The model can be represented as follows:

(1) G=AF+V

where G is the snapshot data vector of the array, V is the noise data vector, F is the spatial signal vector, and A is the N×M dimensional steering matrix of the spatial array, composed of steering vectors $a\;(\theta_{m})$. Each steering vector corresponds to the direction of a signal and can be represented as follows:

$$A=\left[a\left(\theta_{1}\right),a\left(\theta_{2}\right),\ldots ,a\left(\theta_{M}\right)\right]$$

$$\varphi_{0}=\frac{2\pi }{N}$$

Assuming the existence of multiple narrowband signals, represented by plane waves, each signal has a specific amplitude $A_{m}$ and frequency $f_{m}$, as well as incidence angles $\left(\varphi_{m},\theta_{m}\right)$. The signal output of the *q*-th element at a specific moment can be expressed as follows:

$$g\left(q,t\right)=A_{m}\exp\left(j2\pi f_{m}\left(t-\frac{\tau_{q1}}{c}\right)\right)$$

where $\tau_{q1}$ is the spatial delay of element *q* relative to the reference element

$$\tau_{q1}=-R_{0}\sin\theta_{1}\cos\varphi_{q1}$$

Due to the equal azimuth angle spacing of the elements, $\varphi_{q1}$ can be expressed as follows:

(2) $$\varphi_{q1}=q\varphi_{0}-\varphi_{1}$$

The unit vector ${\hat{k}}_{m}$ in the Cartesian coordinate system represents the incidence direction of the *m*-th signal, where $\hat{x}$, $\hat{y}$, and $\hat{z}$ are the unit vectors of the Cartesian coordinate system. The position vector of the *q*-th element can be expressed as follows:

(3) $${\hat{r}}_{q}=\cos\left(q\varphi_{0}\right)\hat{x}+\sin\left(q\varphi_{0}\right)\hat{y}$$

When the signal propagates from the source to the array, due to the different positions of the elements relative to the signal source, there will be slight differences in the time the signal arrives at different elements, resulting in phase differences. We reflect this phase difference using the dot product of the signal direction and the element position vector, which can be used to determine the direction of the signal’s arrival. It can be expressed as follows:

(4) $${\hat{k}}_{m}\cdot {\hat{r}}_{q}=-\sin\theta_{m}\cos\left(q\varphi_{0}-\varphi_{m}\right)$$

For *M* signal sources, the amplitude $A_{m}$ and frequency $f_{m}$ of each signal source can be integrated into a signal model. The steering matrix A(θ,φ) of the circular array can be expressed as follows:

(5) $$A\left(\theta ,\varphi \right)=\left(\begin{array}{ccc} \exp\left(j2\pi R_{0}\cos\left(-\varphi_{1}\right)\sin\theta_{1}\right) & \ldots & \exp\left(j2\pi R_{0}\cos\left(-\varphi_{1}\right)\sin\theta_{M}\right)\\ \vdots & \ddots & \vdots \\ \exp\left(j2\pi R_{0}\cos\left(-\varphi_{N1}\right)\sin\theta_{1}\right) & \ldots & \exp\left(j2\pi R_{0}\cos\left(-\varphi_{NM}\right)\sin\theta_{M}\right) \end{array}\right)$$

where $\varphi_{nm}=n\varphi_{0}-\varphi_{m}$. $\theta_{m}$ is the elevation angle of the *m*-th signal source, $\varphi_{m}$ is the azimuth angle of the *m*-th signal source, and $R_{0}$ is the radius of the circle. We assume $R_{0}=0.5\lambda$, $0^{\circ }⩽\varphi_{m}⩽360^{\circ }$, $0^{\circ }⩽\theta_{m}⩽90^{\circ }$. All angles in this paper use degrees (°) for consistency. Trigonometric functions implicitly convert to radians via $\alpha_{\mathrm{rad}}=\alpha_{\deg}\times \pi /180$.

## 3. The Kalman Filtering Algorithm and MFP Method

### 3.1. Kalman Filtering Algorithm

Kalman filtering (KF) [17] uses a recursive Algorithm, that is, it updates the state parameters based on the prior estimated value of the parameter and the observed value at the current moment. The feature of not needing to store historical information makes it highly efficient. Therefore, in the search and rescue of underwater crashed targets, KF filters the original observation data such as the timing signal, amplitude, and phase collected by the hydrophone from each channel of the array to obtain a denoised signal and makes a preliminary prediction of the signal characteristic parameters. The MVDR Algorithm extracts features for matching field positioning from the signal after Kalman filtering, calculates the copy sound field through the acoustic toolbox, and correlates the copy sound field with the received sound field to obtain a correlation ambiguity map.

The state equation and observation equation of the target are as follows:

(6) X(t)=SX(t−1)+BW(t−1)

(7) Y(t)=HX(t)+V(t)

where *t* is the discrete time, X(t) is the state of the system at time *t*, Y(t) is the observation signal, W(t) is the process noise, V(t) is the observation noise, S is the state transfer matrix, B is the noise driving matrix, and H is the observation matrix.

Assume that W(t) and V(t) are uncorrelated white noises with zero mean and variances Q and R, respectively. The Kalman filter Algorithm’s mathematical expression is as follows:

The state one-step prediction is given by

(8) $$\hat{X}\left(t|t-1\right)=S\left(t-1\right)\hat{X}\left(t-1|t-1\right).$$

The covariance one-step prediction is

(9) $$C\left(t|t-1\right)=S\left(t-1\right)C\left(t-1|t-1\right)S{\left(t-1\right)}^{T}+B\left(t-1\right)Q\left(t-1\right)B{\left(t-1\right)}^{T}.$$

The Kalman filter gain is calculated as

(10) $$t\left(t\right)=C\left(t|t-1\right)H{\left(t\right)}^{T}{\left[H\left(t\right)C\left(t|t-1\right)H{\left(t\right)}^{T}+R\left(t\right)\right]}^{-1}.$$

Then, the state update equation is

(11) $$\hat{X}\left(t|t\right)=\hat{X}\left(t|t-1\right)+t\left(t\right)\left[Y\left(t\right)-H\left(t\right)\hat{X}\left(t|t-1\right)\right],$$

and the state covariance update is

(12) C(t|t)=[I−t(t)H(t)]C(t|t−1).

### 3.2. The Conventional MFP Method

The principle of matching field calculation is to use the ocean environment to establish the ocean model, assuming that the position of the sound source is known. By solving the wave equation and matching field calculation, the sound pressure and other parameters are obtained by correlating them with the receiving field, and the correlation ambiguity diagram is drawn. The correlation’s maximum value is then the sound source’s estimated position. The homogeneous form of the wave equation is

(13) $$\nabla^{2}p-\frac{1}{c^{2}}\frac{\partial^{2}p}{\partial t^{2}}=0$$

In the equation, $\nabla^{2}$ is the Laplacian, *p* is the acoustic pressure, and *c* is the medium sound speed. The normal mode method is used to solve the wave equation. The sound field generated by acoustic wave propagation in the ocean waveguide environment connected to the sea surface above and the seabed below can be decomposed into the superposition of multiple simple normal waves. The key link is to find the modulus depth function. Substituting the modulus depth function into the relationship between the sound pressure field and the modulus depth function, the expression of the sound pressure field can be obtained. Assuming that the sound velocity and the medium density of the sound pressure field are only functions of the depth *Z*, the linear wave equation for a point source in a cylindrical structure is given in the following equation:

(14) $$\frac{1}{r}\frac{\partial }{\partial r}\left(r\frac{\partial p}{\partial r}\right)+\rho \left(z\right)\frac{\partial }{\partial z}\left(\frac{1}{\rho (z)}\frac{\partial p}{\partial z}\right)+\frac{\omega^{2}}{c^{2}\left(z\right)}p=-\frac{\delta \left(r\right)\delta \left(z-z_{s}\right)}{2\pi r}$$

and range equation

(15) $$\frac{1}{r}\frac{d}{dr}\left(r\frac{d\phi }{dr}\right)+k_{rm}^{2}\phi =0$$

By using the method of separating variables and solving the Sturm–Liouville problem [18], the sound pressure field formula of the simple normal mode propagation model can be obtained as follows:

(16) $$p\left(r,z\right)=\sum_{m=1}^{\infty }\Phi_{m}\left(r\right)\Psi_{m}\left(z\right)$$

In the formula, $\Phi_{m}\left(r\right)$ represents the mode distance function of the simple positive wave, and $\Psi_{m}\left(z\right)$ represents the mode depth function of the simple positive wave. The two represent the propagation characteristics of the sound pressure field in the horizontal and vertical directions, respectively. The above equation can be further used to obtain the sound pressure of the sound field at any point in the cylindrical space with distance *r* and depth *z* from the sound source:

(17) $$p\left(r,z\right)\approx \frac{i}{\rho \left(z_{s}\right)\sqrt{8\pi r}}e^{-i\kappa r}\sum_{n=1}^{\infty }\Psi_{n}\left(z_{s}\right)\Psi_{n}\left(z\right)\frac{e^{ik_{zn}r}}{\sqrt{k_{zn}}}$$

In the equation, the asymptotic expansion of the Hankel function is used, and $\rho (z_{s})$ is the medium density at the sound source location.

The AMFP methodology operates through three sequential stages: replica field generation, spatial correlation analysis, and peak localisation. Initially, the Algorithm hypothesises a source position (r,z) and computes the corresponding replica sound field $P_{r}\left(r,z;\omega \right)$ through acoustic propagation modelling. Subsequently, it evaluates the spatial correlation between this replica field and the measured sound field $P(r_{s},z_{s};\omega )$ through cross-spectral analysis, generating an ambiguity surface D(r,z;ω) that quantifies position likelihood:

(18) $$D\left(r,z;\omega \right)=∥P_{r}^{H}\left(r,z;\omega \right)P\left(r_{s},z_{s};\omega \right){∥}_{2}^{2}=P_{r}^{H}\left(r,z;\omega \right)KP_{r}\left(r,z;\omega \right)$$

where the cross-spectral matrix $K\in C^{M\times M}$ is estimated from *L* temporal snapshots:

(19) $$K=\frac{1}{L}\sum_{l=1}^{L}{\tilde{P}}_{l}\left(r_{s},z_{s};\omega \right){\tilde{P}}_{l}^{H}\left(r_{s},z_{s};\omega \right)$$

The constituent vectors are defined as follows:

(20) $$\begin{array}{cc} P_{r}\left(r,z;\omega \right) & ={\left[P_{r1},P_{r2},\ldots ,P_{rM}\right]}^{T}\in C^{M\times 1}\;(\mathrm{Replica}\;\mathrm{field}\;\mathrm{vector}) \end{array}$$

(21) $$\begin{array}{cc} P(r_{s},z_{s};\omega ) & ={\left[P_{1},P_{2},\ldots ,P_{M}\right]}^{T}\in C^{M\times 1}\;(\mathrm{Measured}\;\mathrm{field}\;\mathrm{vector}) \end{array}$$

Here, ${\tilde{P}}_{l}≜P\left(r_{s},z_{s};\omega \right){|}_{t=l\Delta t}$ denotes the lth temporal snapshot of the acoustic measurements, with Δt representing the sampling interval and *L* the total number of statistically independent snapshots.

The AMFP framework optimises detection performance through two sequential computations. First, the MVDR optimal weight vector is derived from

(22) $$w\left(r,z,\omega \right)=K^{-1}P_{r}/\left(P_{r}^{H}K^{-1}P_{r}\right),$$

where $K\in C^{M\times M}$ denotes the estimated cross-spectral matrix and $P_{r}\in C^{M\times 1}$ the replica field vector. The MFP computes the spatial ambiguity function

(23) $$D\left(r,z;\omega \right)=w^{H}Kw,$$

which achieves maximum values at coordinates corresponding to probable source locations. For a horizontal array, it is assumed that the distance of the sound source from the origin is $r_{s}$, the depth of the sound source is $z_{s}$, and the distance of the *n*-th array element from the sound source is $r_{h}$, denoted as $\Gamma_{n}=r_{h}-r_{n}^{0}$. The received Field Vector is

(24) $$p=\Phi A(r_{s},z_{s})$$

In the equation,

$$\Phi =\left[\begin{array}{ccc} \Psi_{1}\left(z_{0}\right)e^{-i\xi_{1}T_{1}} & \ldots & \Psi_{M}\left(z_{0}\right)e^{-i\xi T_{1}}\\ \vdots & \ldots & \vdots \\ \Psi_{1}\left(z_{0}\right)e^{-i\xi_{1}T_{N}} & \ldots & \Psi_{M}\left(z_{0}\right)e^{-i\xi T_{N}} \end{array}\right]$$

$$A={\left[A_{1}\;A_{2}\;\ldots A_{M}\right]}^{T}$$

The matched field vector can be written as $p_{r}=\Phi_{r}A\left(r,z\right)$ In the equation, $\Phi_{r}=\Phi$. Therefore, from the above three formulas, the matched field formula can be obtained as follows:

(25) $$D\left(r,z;\omega \right)=\left|A^{H}\left(r,z\right)\Phi_{r}^{H}\Phi A\left(r_{s},z_{s}\right)\right|$$

Note $\theta =\Phi_{r}^{H}\Phi$ as the normal mode separation matrix. The horizontal array uses the travelling wave property of a simple normal wave, the receiving array has large linearity in the direction of signal arrival, the simple normal wave separation matrix is close to the identity array, and the positioning effect is better. The expression for the transmitted pulse is

(26) $$S\left(t\right)=\left\{\begin{array}{cc} \frac{s_{a}}{2}\sin\left(\omega_{c}t\right)\cdot \left(1-\cos\left(\frac{\omega_{c}t}{4}\right)\right), & \mathrm{for}\;0<t<\frac{4}{f_{c}}\\ 0, & \mathrm{otherwise} \end{array}\right.$$

**Figure 2:**
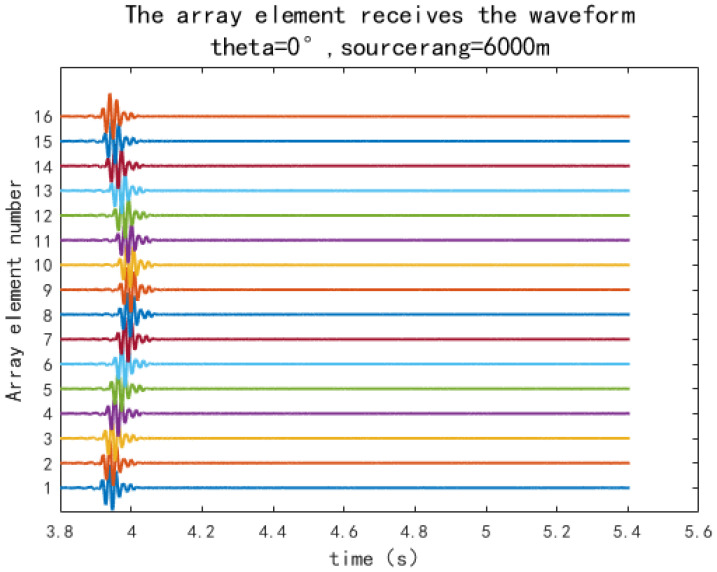
The array element receives the waveform.

In the equation, $f_{c}$ is the pulse centre frequency, $\omega_{c}=2\pi f_{c}$ is the central angular frequency, $s_{a}$ is the pulse peak value, and it is related to the source level $SL=20\log\frac{s_{a}}{p_{\mathrm{ref}}}$, where $p_{\mathrm{ref}}=1\times 10^{-6}\;\mathrm{Pa}$ is the reference sound pressure in water. The centre frequency of the pulse is set to $f_{c}=50\;\mathrm{Hz}$, and the signal bandwidth obtained from the transmitted pulse expression is 50 Hz, which means the signal frequency range is 25 Hz to 75 Hz. Therefore, the Kraken calculation selects the frequency band of 25 Hz to 75 Hz. When the sound source distance is $s_{r}=6\;\mathrm{km}$, and the incident horizontal direction angle is $\theta =0^{\circ }$; the received signal by the array is shown in Figure 2.

## 4. Underwater 3D Source Localisation Based on KF-MVDR-MF Algorithm

In this section, we use a joint Algorithm of DOA estimation and Linear Matched Beamforming Processing methods to locate the three-dimensional coordinates of the target sound source. The marine environment is considered a horizontally uniform and vertically stratified environment. Under such conditions, the normal mode theory [19] has the advantages of fast computation speed and high accuracy. Hence, the normal mode model calculates the ocean acoustic field. The most critical component of the simple normal wave Algorithm involves determining the modulus depth function. Researchers can derive the corresponding acoustic field expression by substituting this function into the established relationship with the sound pressure field. When the seabed parameters are known, the propagation characteristics of the acoustic signal in the seawater can be derived in a simplified way.

The Kalman filter Algorithm is a recursive minimum mean square error estimator, through the recursive prediction and update process, combined with the system dynamic model and real-time observation data to estimate the optimal method of dynamic system state. Its core is to balance the uncertainty between the model prediction and the observed data and gradually approach the true state. Its work is divided into two stages. One is the prediction stage, and the other is the correction stage. The sum of signals and additive noise received by the 16-element hydrophone is first sampled and framed, and then the model parameters of the beacon signal and noise are obtained by parameter estimation, and then the parameters are replaced into the Kalman filter. The enhanced beacon signal of each frame is obtained by Kalman filtering and smoothing, and the final frame is restored. The signal is filtered by Kalman filter to improve the signal-to-noise ratio, and then adaptive beamforming is performed.

AMFP uses conventional beamforming technology to convert data field data and copy field data into beam domain data. It then matches and correlates them in the beam domain to realise the function of sound source localisation. Therefore, matched beam processing is equivalent to matching field processing of the signal in the beam domain. For the vertical linear array, the beam data of the data field $A^{\mathrm{data}}\left(\theta \right)$ and the copy field $A^{\mathrm{rplc}}\left(\theta ,r,z\right)$ are obtained from the corresponding acoustic pressure field as follows:

(27) $$A^{\mathrm{data}}\left(\theta \right)=\sum_{j}e^{-ikz_{j}\sin\theta }p^{\mathrm{data}}\left(z_{j}\right)$$

(28) $$A^{\mathrm{rplc}}\left(\theta ,r,z\right)=\sum_{j}e^{-ikz_{j}\sin\theta }p^{\mathrm{rplc}}\left(z_{j},r,z\right)$$

Among them, $A^{rplc}\left(\theta ,r,z\right)$ represents the acoustic field expression of the copy-field model at depth *z* and distance *r*. $A^{\mathrm{data}}\left(\theta \right)$ represents the acoustic field expression of the actual observed data. $z_{j}$ is the depth of the *j*-th hydrophone, *z* and *r* are the depth of the copy-field sound source and the distance from the vertical array, *k* is the wave number, and θ represents the grazing angle of the sound wave. The objective function of AMFP is defined as follows:

(29) $$B_{L}\left(r,z\right)=\frac{{\left|\int_{\Omega }{\left[A^{rplc}\left(\theta ,r,z\right)\right]}^{*}A^{\mathrm{data}}\left(\theta \right)d\theta \right|}^{2}}{\int_{\Omega }{\left|A^{rplc}\left(\theta ,r,z\right)\right|}^{2}d\theta \times \int_{\Omega }{\left|A^{\mathrm{data}}\left(\theta \right)\right|}^{2}d\theta }$$

In this formula, $B_{L}\left(r,z\right)$ is the objective function used to evaluate the matching degree between the copy-field model and the actual observed data at a given depth *z* and distance *r*. Ω denotes the range of integration over the angles.

**Figure 3:**
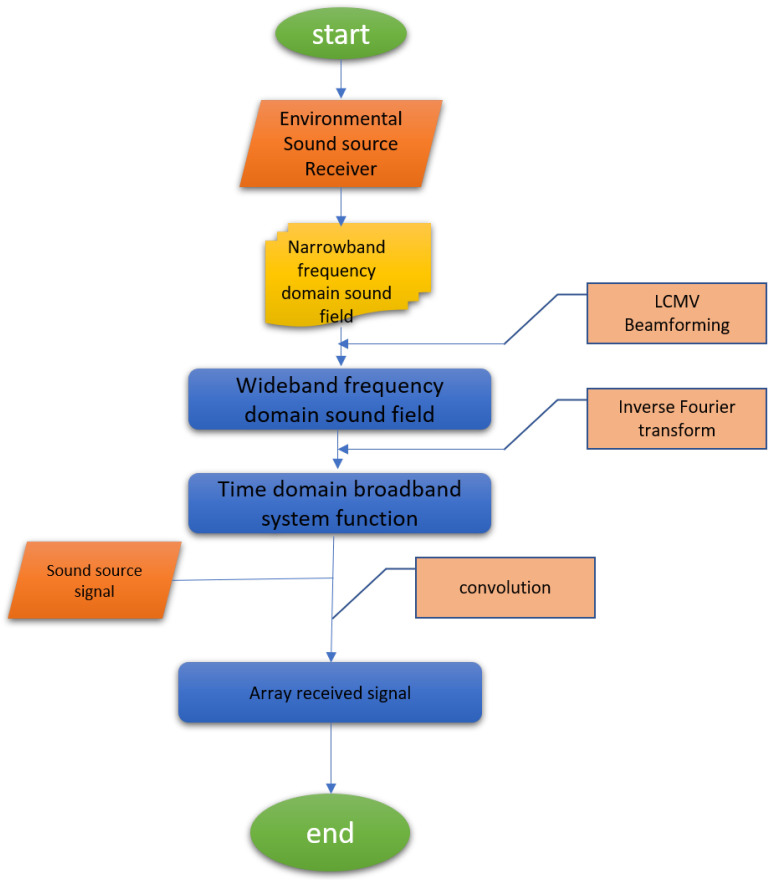
Time domain signal flow chart calculation.

We use Kraken [20] to calculate the ocean sound field. Kraken is a programme within the acoustic toolkit that utilises the normal mode model to compute the ocean sound field. The steps to calculate the time domain signal received by the array are as follows: first, use Kraken to calculate the narrowband frequency domain sound field; then, connect multiple narrowband sound fields to form a wideband sound field; next, use the inverse Fourier transform to obtain the time domain wideband system function; and finally, according to the principle that multiplication in the frequency domain equals convolution in the time domain, convolve the transmitted signal with the system function to obtain the array received signal. The flow chart with Kraken calculating the time domain signal received by the array is shown in Figure 3.

**Figure 4:**
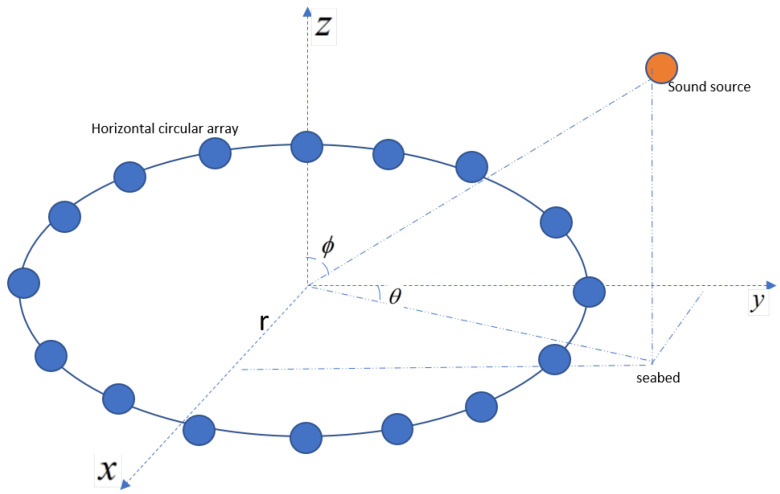
Planar circular array.

The three-dimensional model of the sound field environment is shown in Figure 4. A horizontally uniform circular array with a radius of r=40m and 16 elements is set up, with the geometric centre of the array at (0, 0, 42). The sound source level is set to 200 dB; the source frequency is f=1000Hz, and the source position is (6000,0,25).

The seafloor depth is 42m, the sound speed is $c_{b}=1600\;m/s$, the density is $\rho_{b}=1.5\times 10^{3}\;{\mathrm{kg}/m}^{3}$, and the seafloor attenuation coefficient is $\alpha_{b}=0.2\;\mathrm{dB}/\lambda$, where λ is the wavelength of the incident wave. The sound speed profile of seawater is shown in Table 1, with a density of $\rho =1\times 10^{3}\;{\mathrm{kg}/m}^{3}$ and an attenuation coefficient of α=0.

The environmental parameters are consistent as follows. The sound source is set at a horizontal distance of 6 km from the origin, with the directional angle varying within the range $\theta_{0}\in \left[0^{{}^{\circ}},11^{{}^{\circ}}\right]$, and the signal is set to be a broadband swept-frequency signal with f∈[900Hz,1100Hz]. The details of the KF-MVDR-MF Algorithm are illustrated in Algorithm 1.
**Algorithm 1:** KF-MVDR-MF algorithm for underwater localisation estimation.**Input**: True source position and system parameters, raw acoustic data**Output**: Estimated source location**1** Set the true position of the sound source and parameters;**2** Acquire and read simulation data;**3** Truncate the signal and perform segmented processing;**4** Calculate the number of Fourier-transform points and segmented-processing parameters;**5** Compute the narrowband covariance matrix for the truncated signal;**6** Calculate the energy weighting:$w_{X}\left(f\right)=\frac{{\left|X\left(f\right)\right|}^{2}}{{\sum |X\left(f\right)|}^{2}}$**7** Estimate the covariance matrix:$R_{x}=\frac{1}{N_{\mathrm{tr}}}\sum_{n=1}^{N_{\mathrm{tr}}}R_{x,n}$**8** Compute the sound-field replica vector and the KF-MVDR-MF weight vector:$$\begin{array}{cc} p(r) & =\frac{1}{\rho \sqrt{8\pi r}}\;e^{-j\pi /4}\;\phi \left(z\right)\;\phi^{H}\left(z_{r}\right)\frac{e^{-jkr}}{\sqrt{kr}},\\ w_{C} & =\frac{p(r)}{∥p(r)∥},\\ w_{M} & =\frac{R_{x}^{-1}w_{C}}{w_{C}^{H}R_{x}^{-1}w_{C}}. \end{array}$$**9** Convert results to decibel units:${\mathrm{KF-}\mathrm{MVDR-}\mathrm{MF}}_{\mathrm{dB}}=10\log_{10}\;\left(\mathrm{KF-}\mathrm{MVDR-}\mathrm{MF}\right)$**10** Plot KF-MVDR-MF results and output the estimated sound-source location;

In Algorihm 1 above, $N_{tr}$ represents the number of segments, X(f) represents the Fourier transform of the signal, $R_{x,n}$ represents the covariance matrix of the nth segment, p(r) represents the sound field, ρ represents the density of the medium, ϕ(z) represents the mode shape function, *k* represents the wave number, $w_{C}$ and $w_{M}$ represent the conventional beamforming and beamforming weight vectors, respectively.

## 5. Analysis of Simulation Results

**Figure 5:**
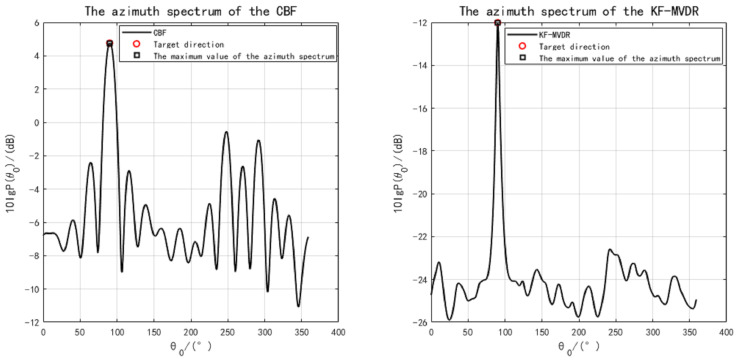
DOA azimuth spectrum comparison at SNR = 10 dB.

When the sound source distance $s_{r}=6$ km and the incident horizontal direction angle is $\theta =90^{\circ }$, DOA azimuth spectrum estimation is performed on the signal with a set SNR of 10 dB, resulting in the bearing spectra for CBF and KF-MVDR as shown in Figure 5. It can be observed that the maximum values of the bearing spectra for both CBF and KF-MVDR are close to the true incident angle, indicating that both methods provide relatively accurate directional estimation. The side lobes of CBF are relatively higher compared to the KF-MVDR method, and the main lobe is wider, suggesting that the Proposed Algorithm has better directional resolution capability. It is worth noting that when the azimuth spectrum scanning angle is $\theta \;=\;90^{\circ }$, the amplitude of the CBF azimuth spectrum is around 5 dB, while the amplitude of KF-MVDR is only around −12 dB, which is a difference of 17 dB. This discrepancy arises because conventional beamforming (CBF) is a relatively simplistic approach, exhibiting limited capability in suppressing interference signals. It will carry out in-phase weighting processing of interference signals and target signals together, resulting in a high response in the azimuth spectrum in the target direction, and there may also be a certain response in other directions, so that the signals in the target direction can interfere with each other. The KF-MVDR method is an adaptive beamforming method which tries to minimise the output power while ensuring the undistorted response to the target signal, so as to suppress the interference signal.

**Figure 6:**
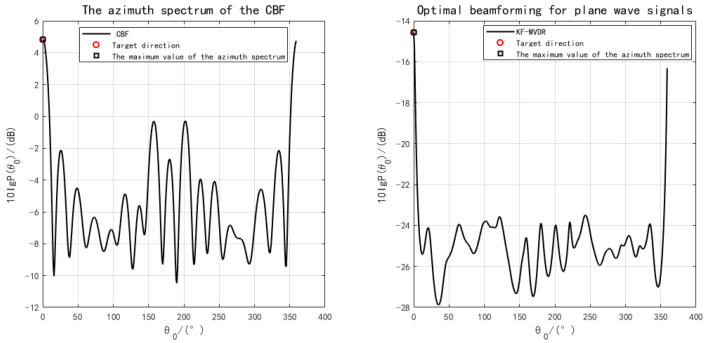
DOA azimuth spectrum comparison at SNR = −20 dB.

With the sound source directional angle set to $\theta_{0}=5^{\circ }$ and SNR = −20 dB, and no errors in the array element positions, the DOA estimation results are shown in Figure 6. As observed, both CBF and MVDR can accurately estimate the direction of the sound source. The simulation parameters satisfy the uniform circular array sampling theorem 2πr/M=15.7m≫λ/2=0.75m, where *r* is the array radius and λ is the wavelength corresponding to the signal’s centre frequency. From the simulation figures, it can be seen that the azimuth spectra of both CBF and KF-MVDR methods are significantly affected by noise. The main maximum intensity of CBF method is lower, and the secondary maximum is more obvious. However, the KF-MVDR method has lower side lobe and performs better in noise suppression.

**Figure 7:**
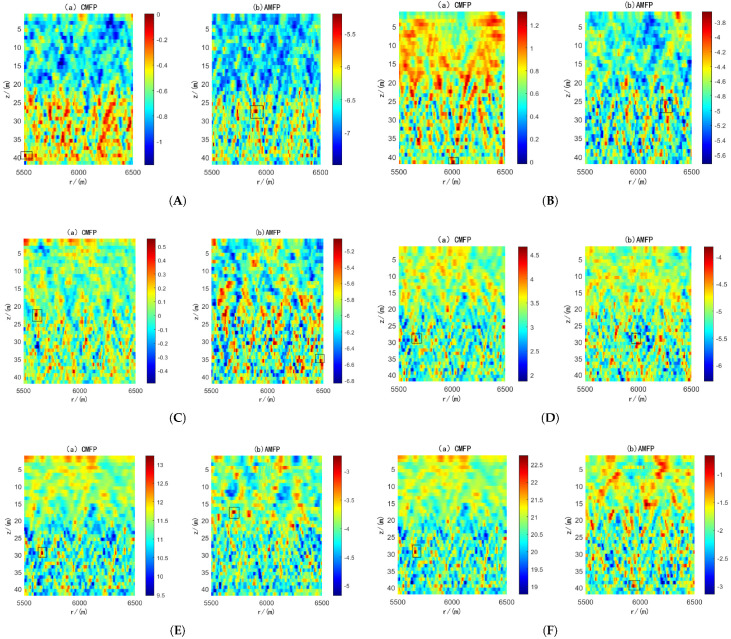
Positioning results under different SNRs. (**A**) SNR = −30 dB; (**B**) SNR = −25 dB; (**C**) SNR = −20 dB; (**D**) SNR = −10 dB; (**E**) SNR = 0 dB; (**F**) SNR = 10 dB.

The environment parameters are set to be consistent with the above simulation, and then the DOA results are input into the MFP method to generate the fuzzy maps of CMFP and AMFP, where the DOA parameters of CMFP are generated by CBF, and the DOA parameters of AMFP are generated by KF-MVDR. The simulation results under different SNR show the performance difference between the two algorithms. This is shown in Figure 7. It can be seen that the estimation results of depth and range are strongly correlated with the estimation accuracy of DOA, and both CMFP and AMFP can accurately estimate depth and range under the premise of accurate DOA estimation. For both CMFP and AMFP methods, the positioning accuracy of the target area shows a gradual improvement trend with the increase in SNR. However, when SNR < −20 dB, the AMFP method can identify the target area more clearly than the CMFP method. The AMFP method has more distinct target region contours and less noise interference, and these results verify the robustness of AMFP in underwater position under challenging noise conditions.

**Figure 8:**
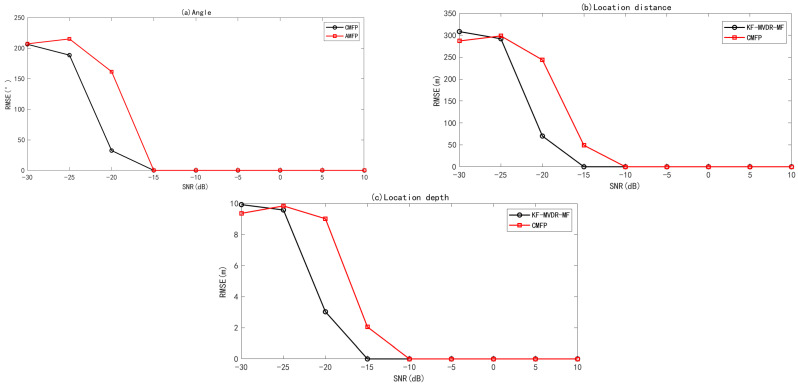
RMSE of joint Algorithm with varying SNR. (**a**) Angle estimation; (**b**) distance estimation; (**c**) depth estimation.

Next, the RMSE of the joint Algorithm concerning angle estimation, positioning distance, and positioning depth was discussed as a function of SNR. One hundred Monte Carlo simulations were performed assuming SNR ∈[−30dB,10dB], sound source orientation angle $\theta_{0}=5^{\circ }$, and no error in array position. The resulting Root mean square error (RMSE) curves for the DOA estimation angle, source depth, and distance as a function of SNR are shown in Figure 8. We can observe that when snr = −10 dB, the RMSE of angle, source depth, and distance approaches 0 for both algorithms, which can achieve perfect 3D position. When the SNR falls below −15 dB, the adaptive joint Algorithm KF-MVDR-MF maintains an RMSE of 0 for angle, source depth, and distance estimation, enabling accurate 3D position of underwater targets even under low-SNR conditions. However, under this SNR condition, the RMSE of the adaptive joint Algorithm KF-MVDR-MF is still 0. The RMSE of angle, source depth, and distance of the linear joint Algorithm CBF-CMFP is larger than that of the adaptive joint Algorithm KF-MVDR-MF. When the SNR is −25 dB, the RMSE of angle, source depth, and distance of the two algorithms reaches the same level again, and neither Algorithm can achieve underwater position well.

**Figure 9:**
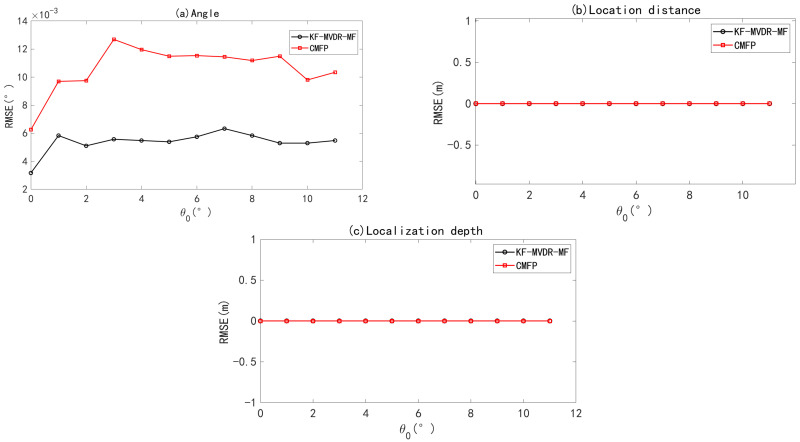
RMSE of joint algorithm with varying $\theta_{0}$. (**a**) Angle estimation; (**b**) distance estimation; (**c**) depth estimation.

The RMSE of the joint Algorithm concerning DOA angle estimation, position distance, and position depth as a function of $\theta_{0}$ is discussed next. The SNR was set to 0 dB, the direction angle of the source was changed within $\theta \in [0^{\circ },11^{\circ }]$, and there was no array position error. Monte Carlo simulation was performed 100 times. The RMSE curves of the DOA estimation angle, source depth, and distance as a function of the source direction angle are shown in Figure 9. As can be seen from Figure 9a, KF-MVDR-MF shows a more stable RMSE of the DOA estimation angle, staying below $5\times 10^{-3}$. However, the RMSE curve of CBF-CMFP fluctuates more and maintains at $10\times 10^{-3}$. Further, from Figure 9b,c, it can be seen that there is no error in the positioning distance and positioning depth between the KF-MVDR-MF and CBF-CMFP Algorithm within the range set by the sound source direction angle.

**Figure 10:**
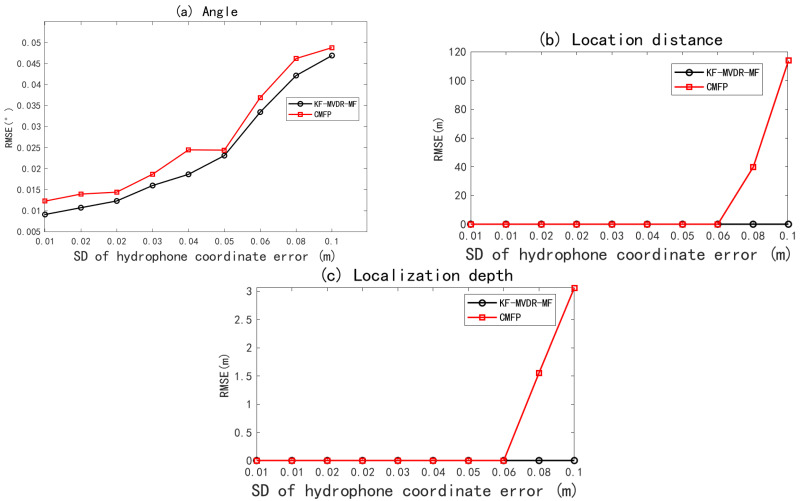
RMSE of joint Algorithm with varying SD of hydrophone coordinate error. (**a**) Angle estimation; (**b**) distance estimation; (**c**) depth estimation.

In the actual underwater environment, the hydrophone installation error is usually between 0.02 m and 0.1 m, affected by the flow disturbance and accuracy limitation. The sensor coordinate error is usually regarded as a random error. It is assumed that the error follows a Gaussian distribution, and the standard deviation can intuitively represent the distribution of the sensor coordinate error. By setting a reasonable standard deviation, the influence of the sensor coordinate error can be simulated, and then its influence on the position accuracy of the sound source can be evaluated. Under Monte Carlo simulations with an incidence angle of 5 and SNR of 0 dB and 100, the RMSE curves for DOA estimation, source depth, and range against hydrophone coordinate error are shown in Figure 10. From Figure 10a, it can be seen that the RMSE of DOA estimation gradually increases with the increase in the array element coordinate error. From Figure 10c, it can be seen that when the standard deviation of the array element coordinate error increases from 0.01 to 0.06, the RMSE of distance and depth of CMFP and KF-MVDR-MF are both 0. As the array element coordinate error continues to increase, the RMSE of distance and depth of CMFP continues to increase, but the RMSE of distance and depth of KF-MVDR-MF is still 0, because KF-MVDR can adaptively adjust the filter gain. The problem of poor resolution in MVDR beamforming system when the array element coordinate error increases is improved by adding a pre-Kalman filter.

## 6. Experimental Data Research

**Figure 11:**
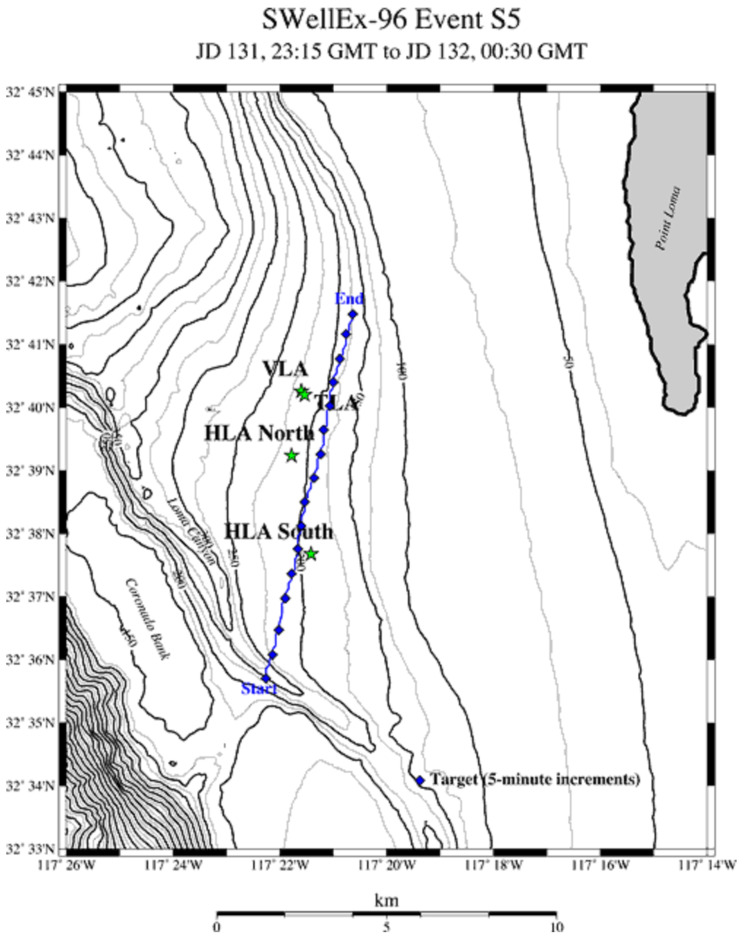
Event S5 bearing track diagram.

**Figure 12:**
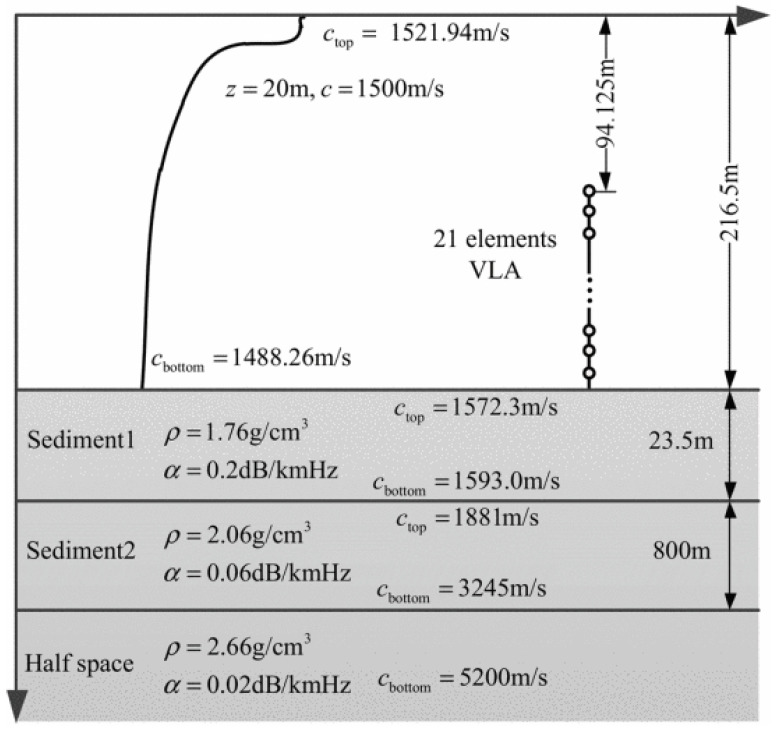
Environmental configuration of SWellEx-96 experiment.

In this section, we used the SWellEx-96 experiment [21] as the data source to evaluate the performance of the proposed method. The SWellEx-96 experiment took place from 10 to 18 May 1996, about 12 km off Point Loma near San Diego, California. The experiment site is characterised by its shallow water environment with a depth of roughly 200 m, a relatively flat seabed, and a downward refracting sound-speed profile. During the SWellEx-96 experiment, event S5 involved a source towed along an isobath. No loud interfering sources were present during this event. The processing frequency band was 50–200 Hz, and the bearing track is shown in Figure 11. The environmental parameters and array configuration are shown in Figure 12. The track of the source vessel (R/V Sproul) began south of all the arrays, proceeding northwards at 5 knots (2.5 m/s). Most of the source-towed track occurred between 180 m and 220 m depths, with the latter part along the 180 m isobath.

**Figure 13:**
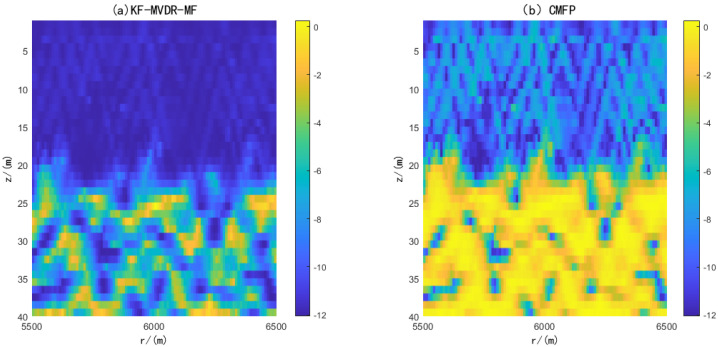
SWellEx-96 experiment Ambiguity Diagram.

Figure 13 shows the performance comparison of the positioning ambiguity map of the proposed method and the CMFP method. It can be seen from the figure that the CMFP Algorithm results have multiple peaks and are relatively scattered, which means that there is a large uncertainty in the position estimation. On the contrary, the proposed KF-MVDR-MF method only has one peak at a horizontal distance of 5 km and a depth of 25 km, indicating that the position of the target can be effectively estimated.

## 7. Conclusions

This paper proposes a 3D underwater position framework based on a horizontal UCA, called the KF-MVDR-MF Algorithm, for deploying a fast response underwater position system in maritime search and rescue scenarios. By combining the Kalman filter-based adaptive beamformer to generate the spatial spectrum for real-time DOA estimation, the adaptive matching field Algorithm uses the DOA information to calculate the range and depth of the targets, to jointly estimate the 3D position of the missing underwater target. The KF-MVDR-MF Algorithm alleviates the angle ambiguity problem and does not require computationally intensive 2D spectral search.

Simulation results show that the positioning error of the proposed method is less than 0.8 m, under the condition of moderate noise with SNR ≤−15 dB. The KF-MVDR-MF Algorithm firstly preprocesses the signal with Kalman filter, and then uses the MVDR matching field Algorithm for positioning, which is suitable for fast signal denoising and feature extraction when the signal noise is large and the quality of array observation data is poor, and improves the accuracy and robustness of single positioning. It is an adaptive joint strategy with enhanced environmental adaptability. The results show that the proposed framework can provide a practical basis for the deployment of rapid response underwater positioning systems in maritime search and rescue scenarios. Although the simulations validated the robustness of the proposed Algorithm in a layered ocean environment, future work could incorporate the seafloor topography into the replica field modelling and construct a seafloor–target coupled acoustic field model to address the environmental mismatch and multi-source interference limitation issues.

## Figures and Tables

**Table 1:** Depth-dependent sound speed profile in seawater.

Depth (*d*) [m]	Sound Speed (*c*) [m/s]
0	1530.0
10	1530.0
15	1527.0
20	1520.0
25	1497.5
42	1497.5

## Data Availability

The SWellEx-96 shallow water experimental data set used in this study is publicly available at: https://swellex96.ucsd.edu/, accessed on 27 June 2025.

## Abbreviations

The following abbreviations are used in this manuscript:

MFP	Matching Field Processing
UCA	uniform circular array
ULA	uniform linear array
MVDR	Minimum Variance Distortionless Response
AMFP	Adaptive Matching Field Algorithm
CMFP	Conventional Matched Field Processing
CBF	Conventional Beamforming
DOA	Direction of Arrival
KF-MVDR-MF	MVDR combined with MFP based on pre-Kalman filtering
